# Plasma cfDNA for the Diagnosis and Prognosis of Colorectal Cancer

**DOI:** 10.1155/2022/9538384

**Published:** 2022-05-31

**Authors:** Zhiwei Wu, Lijiang Yu, Juan Hou, Lihua Cui, Yufeng Huang, Qing Chen, Yan Sun, Wangkun Lu, Chenggong Zhang, Di Sun

**Affiliations:** ^1^Department of Oncology, The Seventh Affiliated Hospital of Yangzhou University, Jingjiang People's Hospital, Taizhou, Jiangsu, China; ^2^Department of Gastrointestinal Surgery, The Seventh Affiliated Hospital of Yangzhou University, Jingjiang People's Hospital, Taizhou, Jiangsu, China

## Abstract

**Objective:**

To evaluate the value of cell-free DNA (cfDNA) for the diagnosis and prognosis of colorectal cancer (CRC).

**Methods:**

Peripheral blood specimens of 120 CRC patients and 90 healthy volunteers (as a control cohort) were extracted. A quantitative real-time polymerase chain reaction (qRT-PCR) was performed to determine the cfDNA expression. Following correlation analyses for cfDNA and clinical endpoints, a receiver operator characteristic (ROC) curve was established to assess the sensitivity and specificity of cfDNA, CEA, VEGF, and CA125 and for evaluating the disease-free survival (DFS) of patients.

**Results:**

The plasma cfDNA level of colorectal cancer patients was significantly higher than that of healthy subjects (*P* < 0.05), and after chemotherapy, cfDNA level was significantly lower than that before chemotherapy (*P* < 0.05). CA125/CEA/VEGF expression significantly correlated with cfDNA level, but not with cfDNA integrity. There was also a significant correlation between tumor differentiation and the cfDNA level. cfDNA has a higher ROC value than the current tumor biomarkers. Survival analysis showed that the DFS of the low cfDNA expression group was longer (29.99 ± 0.78 months) than that of the high cfDNA expression group (27.66 ± 1.05 months, *P*=0.031).

**Conclusion:**

The blood cfDNA is associated with the pathological features of CRC clinical cases and represents a possible indicator for CRC diagnosis and prognosis.

## 1. Introduction

According to the statistical report of the National Cancer Center in 2018, CRC prevalence and death rates in China ranked fourth and fifth, respectively, in tumor morbidity and mortality. In 2017, there were 376,000 novel cases and 191,000 deaths. Both morbidity and mortality have maintained a rapid upward trend, and it has become one of the major life-threatening cancers [1]. The main treatments for CRC are surgery, chemotherapy, and radiotherapy. In recent years, with the advancement of surgery and the extensive development of standardized multidisciplinary treatments and the appearance of new drugs, the therapeutic effect of CRC has been significantly improved. However, about 40% of patients with CRC will have recurrence and metastasis after treatment [2]. Therefore, it is particularly urgent to study the mechanism of CRC development and to find new diagnostic and therapeutic indicators.

Image-based evaluation and biopsy are the main modalities in CRC diagnosis [3]. However, there are many limitations in practical implementation, including challenges in tumor tissue collection, secondary sample collection, and issues of tumor heterogeneity. Liquid biopsy represents a noninvasive or minimally invasive route for detecting circulating tumor cells (CTCs), circulating tumor DNA (ctDNA), circulating free-cell DNA (cfDNA), and exosomes from bodily fluids for tumor diagnostic and prognostic purposes [4]. In comparison to tissue-based biopsy, liquid biopsy has minimum invasive property, leading to enhanced patient compliance and increased reproducibility [5]. Furthermore, cfDNA consists of liberated self-DNA present within blood plasma, being introduced into the bloodstream following cellular necrosis/apoptosis or through active discharge by specific healthy and/or tumor tissues and bearing potential high-consistency genomic details regarding primary tumor composition. Plasma-based cfDNA analysis can be an excellent replacement for a histopathology-based assessment whenever there are challenges regarding tumor biopsy extraction [6]. In addition, blood-based biopsy has increased practicality due to facilitated follow-up assessments. In this study, qRT-PCR was employed to determine cfDNA level in CRC patients and its value in the diagnosis and prognosis of CRC was evaluated.

## 2. Methodology

### 2.1. Sampling

120 primary CRC patients were selected as study subjects from January 2015 to July 2018. Inclusion criteria were as follows: (1) patients were diagnosed by histopathology; (2) case data were recorded intact. (3) KPS score >60 points; and (4) patients were not treated with chemotherapy. Exclusion criteria were as follows: (1) those having concomitant malignant tumor; (2) those having serious conditions, including cardiac, hepatic, and renal conditions; (3) cases having infectious conditions of any nature; and (4) patients suffering from psychiatric conditions leading to patient noncompliance. Of the 120 patients, 68 (56.67%) were male and 52 (43.33%) were female, with no history of chemotherapy; their age ranged from 36 to 78 years, having a mean age of (53.6 ± 6.9) years. All 120 cases were treated with surgery and chemotherapy. Informed written consent was collected from all participants. The study was approved by the Jingjiang People's Hospital Clinical Research Ethics Committee.

### 2.2. Chemotherapy Regimen

The patients received the CAPOX regimen: from the beginning of treatment, capecitabine tablets were orally given to patients with CRC (trade name: Xeloda, Roche Pharmaceutical Co., Ltd., Shanghai, specification 0.5 mg), at dose 1000 mg/m^2^ for 2 times/d, with continuous use for 2 weeks, and on the first day of treatment, an intravenous infusion of oxaliplatin was given (Jiangsu Aosaikang Pharmaceutical Co., Ltd.), at dose 130 mg/m^2^.

### 2.3. Plasma Separation and cfDNA Extraction

Venous blood specimens were placed into EDTA-vacutainers, with plasma collected through centrifugation (1600 g/ten minutes). The supernatant was placed within a fresh tube and underwent centrifugation (16000 g/ten minutes). Purified plasma was slowly extracted without disturbing the lower layer of cells. A 200 *μ*L plasma aliquot was employed for swift DNA extraction, or else placed into −80°C storage. Plasma specimens were ice-thawed followed by centrifugation (10,000 g/180 seconds) prior to DNA purification employing 50 *μ*L elution buffer (QIAamp DNA Blood Mini Kits® Qiagen™, USA) in line with the kit protocols. DNA specimens were finalized for quantification or placed into −20°C storage.

### 2.4. qRT-PCR

qRT-PCR was conducted through the LightCycler LC480® platform (Roche Molecular Systems ™, USA). To determine the cfDNA plasma level, repetitive LINE1- (long interspersed nuclear element 1) 97 bp (both for short and long) and LINE1 300 bp (only for long) DNA fragments were amplified as per previous study protocols [7]. LINE1-97 bp primers allowed amplification for apoptotic and nonapoptotic DNA fragments, while LINE1-300 bp primers allowed amplification solely for nonapoptotic DNA fragments. The global plasma DNA level was reflected through qRT-PCR dataset outcomes using the LINE1 97bp primer. The cfDNA integrity index was obtained through the LINE1 300/LINE1 97 QPCR ratio. A serially diluted, standardized solution containing human genomic DNA (Thermo Fisher Scientific™, USA) was employed as a standard curve for referencing. cfDNA level within individual specimens was determined depending upon the standard curve. qRT-PCR runs were conducted on three independent occasions, employing triplicate for obtaining mean Cq values, consequently utilized for analyses. The reaction mixture volume (per individual qRT-PCR run well) was 20 *μ*L, containing 1 *μ*l DNA template, 0.5 *μ*L forward and reverse primers (LINE1 97 or LINE1 300), 10 *μ*L UltraSYBR Mixture® (Cwbiotech™, China), and 8 *μ*L double-distilled water. The PCR cycle conditions consisted of 60s @ 95°C, followed by 35 cycles of 95°C for 8 seconds/60°C for 15 seconds. Individual plates contained plasma DNA specimens, a negative control (water), and seven serially diluted standardized DNA solutions.

### 2.5. Tumor Biomarker Determination

Electrochemiluminescence was employed for detection. Sera were obtained through centrifuging fasting venous blood, employing an automatic electrochemiluminometer E170® and assorted kits (Roche™, Switzerland). References ranged as follows: cancer antigen CA125 <35 U/mL, carcinoembryonic antigen (CEA) <3.5 ng/mL, neuron-specific enolase (NSE) <16.3 ng/mL, and alpha-fetoprotein (AFP) <7 ng/mL. Postoperative 120 CRC specimens were collected, and the corresponding normal mucosal tissues were used as controls. We used the Western blot assay to detect VEGF/E-cadherin/mTOR/MMP-9 protein expression in tissues.

### 2.6. Statistical Analyses

The cfDNA quantification datasets reflected the mean ± standard deviation (*x* ± SD). A Kruskal–Wallis rank-sum test was employed for comparative analyses. All count datasets were comparatively assessed through the Chi-square test, while measurement datasets were comparatively assessed through the *t*-test. The ROC curve evaluated cfDNA quantification as a screening instrument for CRC clinical cases, while the area under the ROC curve (AUC) was employed for determining accuracy in discerning across two separate conditions for differing essential values. SPSS 21.0 was employed for statistical analyses, and *P* < 0.05 was considered statistical significance.

## 3. Results

### 3.1. Patients' Clinicopathological Characteristics

Based on the TNM staging stipulated by the Union for International Cancer Control (UICC), among 120 patients with CRC, 71 patients (59.17%) were categorized as stages II and III and 49 (40.83%) were stage IV. There were 64 cases (53.3%) of primary tumors in the colon and 56 cases (46.7%) in the rectum; 84 (70%) of the 120 patients belonged to moderately to poorly differentiated CRC; 36 cases (30%) belonged to highly differentiated CRC (Table 1).

**Figure 1:**
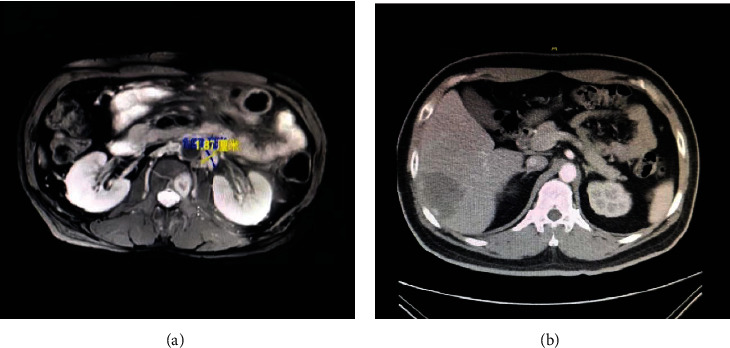
Imaging data for patients with CRC. (a) Rectal cancer and (b) colon cancer.

**Figure 2:**
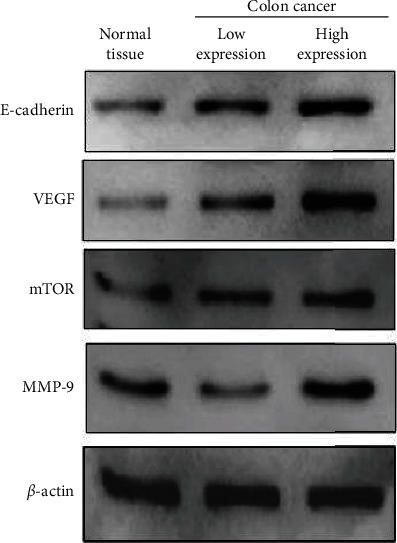
Protein expression levels of E-cadherin, VEGF, mTOR, and MMP-9 in different tissues determined by protein immunoblotting.

The imaging data revealed abnormal thickening and masses in the sigmoid colon. These results suggest that CRC was accompanied by a surrounding infiltration. The walls of the subrectal segments were thickened and strengthened. There were multiple plump lymph nodes around the perirectal artery, mesangial fascia, and right pelvic wall (Figure 1). The results of the Western blot test confirmed the presence of VEGF/E-cadherin/mTOR/MMP-9 overexpression in part of the CRC tissues (Figure 2).

### 3.2. cfDNA Concentration in the Plasma of Healthy Individuals and CRC Patients

**Figure 3:**
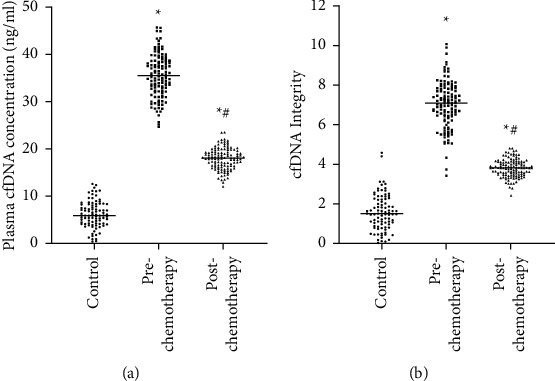
qRT-PCR for quantification of cfDNA concentration and integrity (^*∗*^*P* < 0.05 compared to control group; ^#^*P* < 0.05 compared to prechemotherapy group; each sample was repeated three times).

The qRT-PCR data regarding cfDNA level/quality integrity are illustrated in Figure 1. The cfDNA level in the control cohort stood at 6.18 ± 2.77 ng/mL, while cfDNA integrity reached 1.58 ± 0.90. Prechemotherapy CRC cohort cfDNA level reached 35.51 ± 4.55 ng/mL, while cfDNA integrity was 7.03 ± 1.18. Postchemotherapy CRC cohort cfDNA level was 17.96 ± 2.24 ng/mL, while cfDNA integrity was 3.81 ± 0.45. Prechemotherapy CRC cohort cfDNA level/integrity significantly increased in comparison to postchemotherapy datasets, with both postchemotherapy CRC cohort indexes being significantly rised in comparison to the control cohort, all at *P* < 0.05 (Figure 3).

### 3.3. Associations of cfDNA Level/Integrity and Patients' Clinicopathological Characteristics

Table 2 (cfDNA concentration) and Table 3 (cfDNA integrity) highlight all comparative analysis outcomes. There were no major associations between cfDNA concentration/integrity and gender, age, TNM stage, tumor location, NSE, and AFP expression within the patients' pre/postchemotherapy CRC cohort, all at *P* > 0.05. However, a significant association was identified for CA125/CEA/VEGF expression and cfDNA level, all at *P* < 0.05, while not significantly associated with cfDNA integrity, where *P* > 0.05. There was also a significant association between tumor differentiation and cfDNA level/integrity, where *P* < 0.05 (Tables 2 and 3).

### 3.4. Receiver Operator Characteristic Curve Analyses for cfDNA Levels within CRC Cases

**Figure 4:**
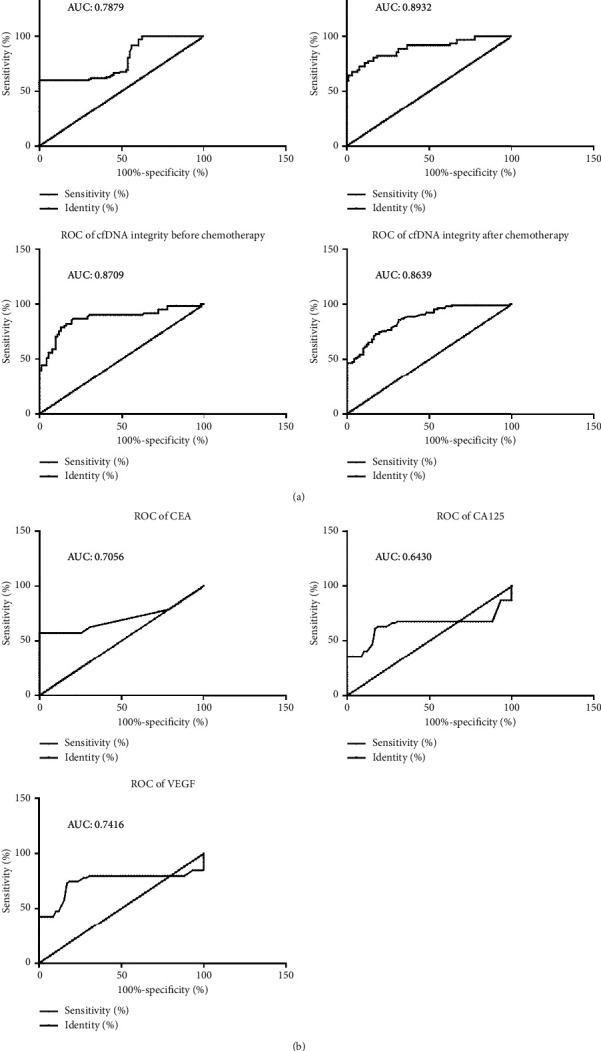
ROC analysis for discrimination between colorectal patients and healthy individuals.

According to the serum cfDNA level of pre/postchemotherapy CRC cohort and tumor biomarker (CEA, CA125, and VEGF) expression profiles, the specificity/sensitivity of these indexes for CRC diagnostic purposes was determined, followed by drawing of corresponding ROC curves (Figure 2). In the CRC cohort, AUCs were as follows: CEA = 0.7056 (95% CI: 0.6308 to 0.7803); CA125 = 0.6430 (95% CI: 0.5351 to 0.7509); VEGF = 0.7416 (95% CI: 0.6412 to 0.8420). In the CRC cohort before chemotherapy, AUCs were as follows: cfDNA level = 0.7879 (95% CI: 0.7253 to 0.8505); cfDNA integrity = 0.8709 (95% CI: 0.8079 to 0.9338). In the CRC cohort after chemotherapy, AUCs were as follows: cfDNA level = 0.8932 (95% CI: 0.8384 to 0.9480); cfDNA integrity = 0.8639 (95% CI: 0.8155 to 0.9124). Based on this, cfDNA level is a reliable tumor screening methodology, with a high sensitivity and specificity in comparison to currently adopted tumor biomarker evaluations (Figure 4).

### 3.5. Survival Analysis

**Figure 5:**
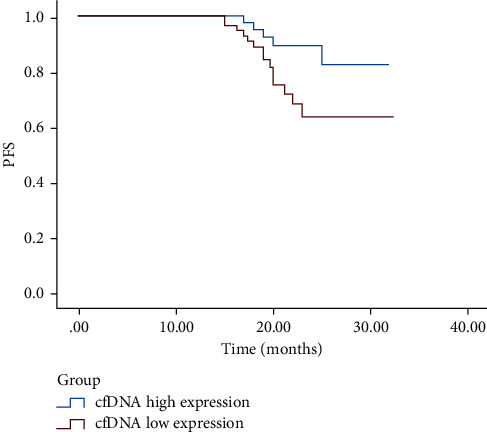
Survivorship curve.

The overall DFS of the 120 CRC cases during this investigation was 29.99 ± 0.78 months. Cases were segregated within two separate cohorts depending upon cfDNA content after treatment; a cfDNA concentration of <18.03 ng/mL was selected in the cfDNA low-expression group, and a cfDNA concentration of ≥18.03 ng/mL was selected in the cfDNA high-expression group. The results showed that the cfDNA low-expression group had a longer DFS (29.99 ± 0.78 months) than the DFS of the cfDNA high-expression group (27.66 ± 1.05 months, *P*=0.031) (Figure 5, Table 4).

## 4. Discussion

Cancer relapse and metastasis are common after tumor resection, radiotherapy, and chemotherapy. Therefore, proper monitoring and identification are critical for cancer management.

Liquid biopsies include blood, urine, saliva, cerebrospinal fluid, and others. The traditional peripheral blood biomarkers, including prostate-specific antigen PSA, carcinoembryonic antigen CEA, alpha-fetoprotein AFP, and cancer antigen CA, are only specific for one or a restricted range of tumors and cannot monitor tumor progression, including onset, treatment, metastasis, and recurrences.

Free DNA, termed as circulating DNA or cell-free DNA (cfDNA), refers to extracellular DNA present in the plasma or serum [8]. As early as 1948, Mandel et al. detected free DNA content within human blood for the first time, but it did not attract the attention of the scientific community at the time [9]. In 1977, Leon et al. first reported higher levels of free DNA within the peripheral blood of cancer patients, and many scholars began to study the relationship between tumors and free DNA [10]. Most of the cfDNA is a double-stranded molecule with a very broad molecular weight between 0.18 kb and 21 kb, and its molecular weight is lower than that of genomic DNA [11]. Within the plasma of healthy individuals, cfDNA comes from apoptotic cells rather than necrotic cells. Furthermore, cfDNA within tumor patient plasma DNA is typically discharged from tumor tissue or as spilled contents from necrotic tumor tissue [8]. The half-life of cfDNA in the blood is about 16 minutes, so cfDNA can be utilized in versatile clinical follow-ups. A number of experiments have shown that the liver, kidney, and spleen are involved in the rapid clearance of cfDNA. cfDNA could be identified in the plasma/serum specimens from both cancer patients and patients with other conditions, as well as healthy volunteers [12]. Previously, reliable results could not be obtained from minute levels of cfDNA within healthy human plasma because of the low sensitivity of analytical methods. The development of modern cutting-edge biotechnology, such as qRT-PCR or fluorescent dyes, can detect cfDNA within healthy blood, making it possible to monitor cfDNA in healthy and subhealthy populations. The concentration of cfDNA in cancer patients is typically upregulated, as validated through multiple investigations [13]. Furthermore, several other investigations validated the role of cfDNA in the prognosis of tumor relapse and patient survival and in evaluating therapeutic responses. The cfDNA concentration is higher in many cancer patients and can be used as an independent risk variable in survival odds [14]. Hsieh et al. highlighted that upregulated cfDNA levels typically suggest tumor relapse in postoperative esophageal cancer patients [15]. This method was more sensitive than CEA or imaging diagnostic methods and has high specificity and sensitivity.

A number of investigations revealed that cfDNA integrity could augment the accuracy of cfDNA identification [16]. cfDNA comes from apoptotic and/or necrotic cells. The size of cfDNA fragments released from apoptotic cells is similar in the range of 185–200 bp due to enzymatic hydrolysis. An increased proportion of cfDNA is within tumor patient plasma derived from necrotic cells [17]. The length distribution of DNA fragments released by these cells differs from healthy individuals, and bioinformatics-based analyses could predict tumor burden based on the cfDNA levels.

CEA, CA125, and other tumor markers were significantly correlated with clinical pathological features of CRC patients, although such specificity is low [18]. Histopathology remains a gold standard for CRC diagnosis, although it does not help with early diagnosis.

Recently, a number of studies demonstrated that the level and integrity of cfDNA could serve as suitable biomarkers for the diagnosis and prognosis of tumors [19]. In malignant solid tumors, DNA integrity is linked to tumor burden and clinical outcome [20]. The DNA integrity index increased in breast cancer patients in comparison to benign tumor/healthy control cohorts, and increased DNA integrity led to reduced odds of relapse [21]. In CRC and hepatocellular cancer patients, DNA integrity was extremely upregulated [22]. Consequently, increased fragmentation of cfDNA was suggested to justify such findings in malignant solid tumors.

Our research was the pioneer quantitative analysis of cfDNA within nondiseased individuals and CRC cases before and after chemotherapy. Our results demonstrated that the cfDNA level in CRC cases was significantly higher than in healthy controls. Interestingly, the cfDNA level was not significantly linked to gender, age, TNM stage, tumor location, or AFP/NSE expression, but it was significantly correlated with CEA and CA125 expression. In addition, there was an association between tumor differentiation and cfDNA level/integrity. This study revealed that the cfDNA level was different in samples prior to and after chemotherapy, suggesting that cfDNA could be a biomarker for CRC therapeutic effectiveness.

As a means of evaluating the role that cfDNA plays in CRC screening, our research compared the specificity and sensitivity of cfDNA and three other tumor biomarkers (CEA, CA125, and VEGF), followed by ROC curve analysis. The latter validated that the AUC for cfDNA is larger than CEA, VEGF, and CA125 in postchemotherapy cases, indicating that the diagnostic effect of cfDNA as a diagnostic parameter in CRC is better than that of CEA, VEGF, and CA125 and could be an alternative methodology for CRC diagnosis and prognosis. Further exploring the relationships between cfDNA expression levels and prognosis, we found that patients with lower cfDNA levels had a longer DFS, suggesting that cfDNA level could be a prognostic marker for patients with CRC.

The clinical deployment of cfDNA is a novel offer in a fast-emerging research niche [22]. For such a technology to be implemented within the practical clinical setting, there are obstacles that require circumvention. Such obstacles include the lack of standardized operating procedures (SOPs) regarding cfDNA analyses—typically encompassing the storage and handling of such samples as well and ultimately influencing the quality and effectiveness of such analytical methods. Thus, standardized guidelines should be implemented, dictating all factors regarding cfDNA analytical protocols, prior to initiating multicenter clinical trials, together with the definition of relevant positive and negative controls for reducing the risk of incurring false-negative or -positive results.

This study demonstrates the association between cfDNA and pathological characteristics of patients with CRC, as well as the effectiveness of cfDNA as a diagnostic indicator for CRC, as a proof of concept for cfDNA deployment for such diagnostic and prognostic purposes. Because of its characteristics of noninvasiveness and rapid accessibility, cfDNA has great potential as an indicator for tumor diagnosis and monitoring.

## Figures and Tables

**Table 1:** Demographic and clinical features of patients.

Variables	*N*/*x* ± SD	%
Gender		
Male	68	56.67
Female	52	43.33
Age		
>65	34	28.33
≤65	86	71.67
Tumor site		
Colon	64	53.33
Rectum	56	46.67
TNM stage		
II/III	71	59.17
IV	49	40.83
Tumor differentiation		
Low-medium	88	73.33
High	32	26.67
E-cadherin		
High expression	68	56.67
Low expression	52	43.33
VEGF		
High expression	75	62.5
Low expression	45	37.5
mTor		
High expression	84	70.00
Low expression	36	30.00
MMP-9		
High expression	73	60.83
Low expression	47	37.17
CA125 (U/ml)	3.08 ± 7.88	—
CEA (ng/mL)	3.15 ± 0.76	—
NSE (ng/mL)	8.24 ± 6.72	—
AFP (ng/mL)	5.10 ± 0.91	—

**Table 2:** Correlation between total plasma cfDNA level and clinical characteristics.

Variables	Before chemotherapy	After chemotherapy
Gender		
Male	35.47 ± 5.39	17.83 ± 2.28
Female	36.26 ± 4.36	18.35 ± 1.98
*P*	0.3901	0.1928
Age		
>65	36.84 ± 5.42	18.09 ± 2.10
≤65	35.49 ± 4.71	17.86 ± 2.27
*P*	0.1781	0.6106
Tumor site		
Colon	35.79 ± 4.92	18.16 ± 2.21
Rectum	35.39 ± 4.43	17.70 ± 2.12
*P*	0.6426	0.2487
TNM stage		
II/III	36.03 ± 4.19	17.99 ± 2.13
IV	35.71 ± 5.97	18.25 ± 2.39
*P*	0.7306	0.5331
Tumor differentiation		
Low-medium	35.48 ± 3.87	18.75 ± 1.90
High	33.16 ± 4.05	16.89 ± 2.02
*P*	**0.0049**	<**0.0001**
CA125		
≥35 U/ml	37.16 ± 3.92	18.73 ± 1.97
<35 U/ml	34.62 ± 4.99	17.40 ± 2.08
*P*	**0.0048**	**0.0009**
CEA		
≥3.5 ng/mL	37.90 ± 5.09	18.90 ± 2.12
<3.5 ng/mL	35.52 ± 4.35	17.52 ± 2.03
*P*	**0.0128**	**0.0006**
NSE		
≥16.3 ng/mL	36.70 ± 3.87	18.58 ± 2.33
<16.3 ng/mL	35.90 ± 5.14	18.25 ± 2.34
*P*	0.6494	0.6847
AFP		
≥7 ng/mL	34.72 ± 4.75	18.08 ± 2.25
<7 ng/mL	36.02 ± 5.08	17.57 ± 0.94
*P*	0.5756	0.2725
E-cadherin		
High expression	35.38 ± 4.84	17.72 ± 2.28
Low expression	35.42 ± 5.32	18.32 ± 2.21
*P*	0.9645	0.1504
VEGF		
High expression	37.09 ± 4.50	19.95 ± 1.83
Low expression	35.41 ± 4.32	18.43 ± 2.06
*P*	**0.0468**	<**0.0001**
mTor		
High expression	35.82 ± 4.85	18.12 ± 1.65
Low expression	35.80 ± 4.49	18.45 ± 2.24
*P*	0.9832	0.4822
MMP-9		
High expression	36.60 ± 4.87	17.83 ± 2.04
Low expression	35.17 ± 5.01	18.13 ± 1.99
*P*	0.1232	0.4289

Bold values mean *P* < 0.01.

**Table 3:** Correlation between integrity of cfDNA and clinical characteristics.

Variables	Before chemotherapy	After chemotherapy
Gender		
Male	6.96 ± 1.14	3.78 ± 0.40
Female	6.95 ± 1.24	3.81 ± 0.42
*P*	0.9635	0.6911
Age		
>65	7.09 ± 1.20	3.86 ± 0.40
≤65	7.27 ± 1.37	3.83 ± 0.41
*P*	0.5037	0.7168
Tumor site		
Colon	6.94 ± 1.34	3.83 ± 0.40
Rectum	7.13 ± 1.25	3.86 ± 0.45
*P*	0.4430	0.6997
TNM stage		
II/III	7.15 ± 1.33	3.83 ± 0.47
IV	6.92 ± 1.14	3.85 ± 0.43
*P*	0.1105	0.8130
Tumor differentiation		
Low-medium	7.38 ± 0.99	3.88 ± 0.40
High	6.31 ± 1.30	3.56 ± 0.47
*P*	<**0.0001**	**0.0003**
CA125		
≥35 U/ml	7.26 ± 1.97	4.12 ± 0.39
<35 U/ml	6.85 ± 1.21	3.98 ± 0.37
*P*	0.2178	0.0573
CEA		
≥3.5 ng/mL	7.80 ± 1.17	3.85 ± 0.45
<3.5 ng/mL	7.74 ± 1.09	3.76 ± 0.43
*P*	0.7831	0.2881
NSE		
≥16.3 ng/mL	7.41 ± 1.27	3.74 ± 0.35
<16.3 ng/mL	7.65 ± 1.01	3.84 ± 0.41
*P*	0.3333	0.1857
AFP		
≥7 ng/mL	7.56 ± 1.08	3.83 ± 0.44
<7 ng/mL	7.34 ± 1.21	3.94 ± 0.52
*P*	0.3363	0.2481
E-cadherin		
High expression	7.07 ± 1.27	3.81 ± 0.50
Low expression	7.05 ± 1.26	3.91 ± 0.48
*P*	0.9381	0.3267
VEGF		
High expression	7.18 ± 1.32	3.84 ± 0.41
Low expression	6.96 ± .1.14	3.90 ± 0.36
*P*	0.2948	0.4550
mTor		
High expression	7.05 ± 1.22	3.84 ± 0.48
Low expression	7.08 ± 1.38	3.91 ± 0.35
*P*	0.9058	0.4317
MMP-9		
High expression	7.05 ± 1.31	3.86 ± 0.44
Low expression	7.07 ± 1.17	3.81 ± 0.43
*P*	0.9324	0.5410

Bold values mean *P* < 0.01.

**Table 4:** Survival time of patients in different groups.

Subject	Time (months)	95% CI		*P*
		Upper limit	Lower limit	
cfDNA high expression	29.99 ± 0.78	28.46	31.54	0.031
cfDNA low expression	27.66 ± 1.05	25.60	29.72	
Total	28.92 ± 0.71	27.53	30.31	

## Data Availability

The shared data can be made available from the corresponding author (jjsundijj@163.com).
